# Polydopamine Coating of Graphitic Carbon Nitride, g-C_3_N_4_, Improves Biomedical Application

**DOI:** 10.3390/biomedicines12061151

**Published:** 2024-05-23

**Authors:** Mehtap Sahiner, Sahin Demirci, Nurettin Sahiner

**Affiliations:** 1 Department of Bioengineering, Faculty of Engineering, Canakkale Onsekiz Mart University Terzioglu Campus, 17100 Canakkale, Turkey; sahinerm78@gmail.com; 2 Department of Chemistry, Faculty of Sciences, Canakkale Onsekiz Mart University Terzioglu Campus, 17100 Canakkale, Turkey; sahindemirci@gmail.com; 3 Department of Ophthalmology, Morsani College of Medicine, University of South Florida, 12901 Bruce B Downs B. Downs Blv., MDC 21, Tampa, FL 33612, USA

**Keywords:** graphitic carbon nitride (g-C_3_N_4_), polydopamine coating, photoactive composite

## Abstract

Graphitic carbon nitride (g-C_3_N_4_) is an intriguing nanomaterial that exhibits photoconductive fluorescence properties under UV–visible light. Dopamine (DA) coating of g-C_3_N_4_ prepared from melamine was accomplished via self-polymerization of DA as polydopamine (PDA). The g-C_3_N_4_ was coated with PDA 1, 3, and 5 times repeatedly as (PDA@g-C_3_N_4_) in tris buffer at pH 8.5. As the number of PDA coatings was increased on g-C_3_N_4_, the peak intensity at 1512 cm^−1^ for N–H bending increased. In addition, the increased weight loss values of PDA@g-C_3_N_4_ structures at 600 °C from TGA thermograms confirmed that the coating was accomplished. The band gap of g-C_3_N_4_, 2.72 eV, was reduced to 0.87 eV after five coatings with PDA. A pristine g-C_3_N_4_ was found to have an isoelectric point (IEP) of 4.0, whereas the isoelectric points of 1PDA@g-C_3_N_4_ and 3PDA@g-C_3_N_4_ are close to each other at 3.94 and 3.91, respectively. On the other hand, the IEP of 5PDA@g-C_3_N_4_ was determined at pH 5.75 assuming complete coating with g-C_3_N_4_. The biocompatibility of g-C_3_N_4_ and PDA@g-C_3_N_4_ against L929 fibroblast cell lines revealed that all PDA@g-C_3_N_4_ coatings were found to be biocompatible up to a 1000 mg/mL concentration, establishing that PDA coatings did not adversely affect the biocompatibility of the composite materials. In addition, PDA@g-C_3_N_4_ was screened for antioxidant potential via total phenol content (TPC) and total flavonoid content assays and it was found that PDA@g-C_3_N_4_ has recognizable TPC values and increased linearly with an increased number of PDA coatings. Furthermore, blood compatibility of pristine g-C_3_N_4_ is enhanced considerably upon PDA coating, affirmed by hemolysis and the blood clotting index%. Additionally, α-glucosidase inhibitory properties of PDA@g-C_3_N_4_ structures revealed that 67.6 + 9.8% of this enzyme was evenly inhibited by 3PDA@g-C_3_N_4_ structure.

## 1. Introduction

Graphitic carbon nitrides (g-C_3_N_4_) are fluorescent polymeric materials made of C and N elements. They are nanomaterials with photocatalytic properties, high physicochemical stability, tunable optical properties, controllable band gap and band position, and low toxicity [1,2,3,4,5,6]. It is envisaged that g-C_3_N_4_ can be used for pH-sensitive drug delivery systems as a therapeutic agent in both modeling and applied sciences. There are studies on the use of g-C_3_N_4_ as a nanocarrier for anticancer drugs such as lonidamine [7], carboplatin [8] and/or cardiovascular drugs such as levosimendan [9] and curcumin [10,11]. Therefore, most of these studies concluded that g-C_3_N_4_-based nanomaterials can be used as pH-sensitive drug carriers in cancer imaging and therapy [12]. Despite its biocompatibility and biometabolizability properties, g-C_3_N_4_ also has some shortcomings. These include low solubility in water, a relatively large particle size, a low electrical conductivity, and a lack of absorption of visible light above 460 nm [2]. Since g-C_3_N_4_ attracts great attention due to its high chemical and physical stability as well as its promising photocatalysts [13,14,15], it can be used, for example, in photocatalysis for the degradation of organic pollutants in wastewater [10]. The tunable band structure of g-C_3_N_4_s is another plus feature for wide ranging applications.

Dopamine (DA), (3,4-dihyroxyphenyl)ethylamine and its’ polymeric forms as poly(3,4-dihyroxyphenyl) ethylamine (p(DA)) has many different biological activities [16]. Due to its catechol groups, it has antioxidant, adhesive [17], and enzyme inhibiting properties [18]. The synthesis of DA occurs via a biochemical pathway that involves the amino acid tyrosine [19]. DA is one of the most important neurotransmitters in the human body and provides transmission of signals between nerve cells, and a deficiency of DA can cause many neurological and mental diseases [20]. It was reported that through the functionalization of polydopamine nanoparticles, an electrochemical aptasensor was developed for the rapid, accurate, and economical detection of glycated albumin as a promising biomarker for glycemic management [21]. There are also reports in the literature about the use of DA as a medication [7,22]. By coating g-C_3_N_4_ with PDA, g-C_3_N_4_ can be gained with new and intriguing properties. Here, DA is used for the coating of g-C_3_N_4_ multiple times, e.g., 1, 3, and 5 times as 1PDA@g-C_3_N_4_, 3PDA@g-C_3_N_4_, and 5PDA@g-C_3_N_4_, respectively, and their characterization was carried out as well as an evaluation of their biomedical use. The enzyme α-glucosidase inhibition capacity, the cytotoxicity, as well as the antioxidant properties of PDA-coated g-C_3_N_4_ were investigated. Thus, the potential for use in the biomedical field of composite structures obtained by the PDA coating of g-C_3_N_4_ was determined.

## 2. Materials and Methods

### 2.1. Materials

To synthesize the graphitic carbon nitride (g-C_3_N_4_)-based structures, melamine (99%, Sigma-Aldrich, St. Louis, MO, USA) was used as a precursor. Dopamine hydrochloride (DA, 98%, Sigma-Aldrich) was used as received. The coating of g-C_3_N_4_ with PDA was carried out in tris buffer (99%, Sigma Aldrich). Folin Ciocalteu’s phenol reagent (Sigma-Aldrich, 99%), sodium nitrite (NaNO_2_, +97%, Acros, Fukuoka, Japan) and aluminum chloride (AlCl_3_, 99.9%, Alfa-Aesar, Haverhill, MA, USA) were used in antioxidant tests. The enzyme α-Glucosidase, from *Saccharomyces cerevisiae* (*S. cerevisiae*) (Sigma-Aldrich), and substrate p-nitrophenyl-α-D-glucopyranose (≥99%, Sigma-Aldrich) were used in the enzyme inhibition tests. Gram-negative bacteria *Escherichia coli* (*E. coli*) (ATCC 8739) and Gram-positive bacteria, *Staphylococcus aureus* (*S. aureus*) (ATCC 6538) were obtained from KWIK-STIK™ Microbiologics (St. Cloud, MN, USA) for antibacterial activity tests. Growth mediums for bacteria were nutrient agar (NA) and nutrient broth (NB), which were obtained from BD DifcoTM (Becton, Dickinson and Company, Sparks, MD, USA).

### 2.2. Synthesis of g-C_3_N_4_ and Its PDA Coating

The synthesis of graphitic carbon nitride (g-C_3_N_4_) was carried out according to the procedures reported in the literature with some modifications [23,24,25]. Briefly, 10 g of melamine was added to a porcelain crucible and placed in a furnace and the furnace was heated to 550 °C with 3 °C/min heating rate and kept at 550 °C for 4 h. The porcelain crucible was then cooled to room temperature and the yellowish solid obtained was ground in a porcelain mortar with a pestle and dispersed in 500 mL of water. Then, the suspension of g-C_3_N_4_ was sonicated for 5 h with stirring every 1 h. Finally, the dispersed g-C_3_N_4_ was collected by centrifugation at 10,000 rpm for 10 min and was dried in a freeze dryer (Alpha 2-4 LSC, Christ, Osterode am Harz, Germany) at −80 °C, and stored in closed tubes for further use.

The coating of g-C_3_N_4_ with polydopamine (PDA) was realized by self-polymerization of DA in tris buffer [26]. Accordingly, 4.5 g of washed and dried g-C_3_N_4_ was suspended in 450 mL of 10 mM tris buffer at pH 8.5 solution and sonicated for 3 h at 20 °C to ensure the exfoliation of g-C_3_N_4_ layers. Next, a freshly prepared 50 mL 20 mg/mL DA solution in 10 mM tris buffer at pH 8.5 was added to this solution and stirred at 1000 rpm mixing rate at room temperature. The final concentration of DA was 2 mg/mL in 500 mL 10 mM tris buffer at pH 8.5. Then, the self-polymerization of DA was carried out in the presence of g-C_3_N_4_ at 1000 rpm mixing rate for 6 h to coat g-C_3_N_4_ with PDA. Finally, PDA-coated g-C_3_N_4_s (1PDA@g-C_3_N_4_) were collected by centrifugation at 10,000 rpm for 10 min and washed twice with DI water to remove impurities from the medium. The yellowish color of g-C_3_N_4_ changed to a brownish color by coating with PDA. Then, 1PDA@g-C_3_N_4_ (1.5 g) was separated, and the remaining 3 g of 1PDA@g-C_3_N_4_ continued to be coated with PDA by applying the same coating procedure. This coating process was carried out 5 times in a row and after each two-coating process, 1.5 g of PDA@g-C_3_N_4_ was dried and separated. The brownish color of 1PDA@g-C_3_N_4_ turned black after the 5th coating with PDA. The washed and dried PDA-coated g-C_3_N_4_ structures were labelled as 1PDA@g-C_3_N_4_, 3PDA@g-C_3_N_4_, and 5PDA@g-C_3_N_4_ structures and stored in closed tubes for further use.

### 2.3. Characterization of PDA@g-C_3_N_4_

The functional group analysis of the g-C_3_N_4_ and PDA@g-C_3_N_4_ structures was performed using Fourier transform infrared spectroscopy (FT-IR) (Nicolet iS10, Thermo-Scientific, Waltham, MA, USA) in the range of 650–4000 cm^−1^, with an attenuated total reflection (ATR) technique, in 4 repetitions.

Thermal stabilities of the g-C_3_N_4_ and PDA@g-C_3_N_4_ structures were compared by a thermogravimetric analyzer (TGA, SII TG/DTA6300, Exstar, Seiko Instruments Corp., Tokyo, Japan). Thermogravimetric analyses were carried out by heating the samples weighing 3–5 mg (PDA@g-C_3_N_4_ samples) from 100 °C to 600 °C with a temperature increase of 10 °C/min under 200 mL/min N_2_ gas flow.

Zeta potential measurements of the g-C_3_N_4_ and PDA@g-C_3_N_4_ structures were carried out by suspending them in 1 mM KNO_3_ solutions at 1 mg/mL concentration using a zeta potential analyzer (Zeta-Pals, BIC, New York, NY, USA). Zeta potential measurements for ionic-group-containing materials (–OH, NH_2_, –COOH, –SO_3_H, etc.) are generally carried out in a KNO_3_ solution that has constant pH variation to prove that the solution is an inert electrolyte [27]. In addition, the isoelectric point values of the materials were calculated by measuring the zeta potential values of the g-C_3_N_4_ and PDA@g-C_3_N_4_ structures at different pH values of material suspension solutions. Furthermore, the zeta potential of the g-C_3_N_4_ and PDA@g-C_3_N_4_ structures was examined at different concentrations of KNO_3_ solution, 0.0, 1, 10, 100 mM, to determine the effect of KNO_3_ salt.

X-ray diffraction (XRD) pattern analysis of the g-C_3_N_4_ and PDA@g-C_3_N_4_ structures by a Cu K_α_ X-ray source (40 kV, 40 mA) at a wavelength of 1.5418 Å with a scanning 2θ range of 20°–70° was performed using an XRD analyzer (XDR, Bruker D8 Advance Diffractometer, Billerica, MA, USA) with CuK_α_ radiation.

The optical diffuse reflectance of the g-C_3_N_4_-based materials was measured at room temperature via a UV–VIS–NIR spectrometer (UV-3600, Shimadzu, Kyoto, Japan) operating in the 200 to 2000 nm range. BaSO_4_ was used to obtain a reference of non-absorbing reflectance. The generated reflectance-versus-wavelength data were used to estimate the material’s bandgap by converting it to absorbance data using the Kubelka–Munk Equation (1):

α/S = (1 − R)^2^/2R (1)

where R is the reflectance, and α and S are the absorption and scattering coefficients, respectively [28,29].

The UV–Vis spectrum (UV-Vis spectrometer, Genesys 180, Thermo Scientific) of the g-C_3_N_4_-based structures was recorded between 200 and 800 nm for the samples of 0.02 mg/mL concentrations of g-C_3_N_4_s. Before UV–Vis spectroscopy measurements, the prepared g-C_3_N_4_-based structure solutions in water were sonicated for 2 h to exfoliate the structures and obtain a good suspension. A fluorescence spectrometer (Spectrofluorometer FS5, Edinburgh Instruments, Livingston, UK) was used to determine the optical properties of the prepared g-C_3_N_4_ structures. The fluorescence properties of g-C_3_N_4_ and PDA@g-C_3_N_4_ mixtures prepared at a 0.2 mg/mL concentration in water were examined at a 325 nm excitation wavelength and 700 PMT V between 330 and 600 nm in wavelength range.

Moreover, the QY% values for the g-C_3_N_4_-based structures were calculated via following the literature [30]. Using quinine sulfate as a reference, the quantum yield% (QY%) values of the g-C_3_N_4_-based structures were computed. Quinine in 0.5 M H_2_SO_4_ was used as a control, with a QY value of 54% and an excitation wavelength of 345 nm. The QY% values of the g-C_3_N_4-_based structures were calculated using the Equation (2).

QY% = QY%_std_ (I/I_std_) · (OD_std_/OD) · (η^2^/η_std_^2^) (2)

where ‘QY’ is fluorescence quantum yield, ‘I’ is the integrated fluorescence intensity, ‘OD’ is the UV–vis absorbance, and ‘η’ is the refractive index of the solvents used for the g-C_3_N_4_-based structure suspension solutions, water (η = 1.333) and 0.5 M H_2_SO_4_ in water (η = 1.346).

### 2.4. Biomedical Properties of PDA Coated g-C_3_N_4_

#### 2.4.1. Cytotoxicity and Blood Compatibility of PDA-Coated g-C_3_N_4_

Biocompatibility and hemocompatibility tests were performed according to the literature [31], and details are given in the Supplementary Material.

#### 2.4.2. Antioxidant Activity Assays for PDA-Coated g-C_3_N_4_

In the determination of antioxidant capacity and enzyme inhibition tests of g-C_3_N_4_ and PDA@g-C_3_N_4_, samples weighing 2000 mg/mL were suspended in DI water and these mixtures were used for α-glucosidase enzyme inhibition, total phenol content (TFC), and total flavonoid content (TFC) tests. The α-glucosidase inhibition test was performed by modifying the method in the literature [26]. A sample solution of 700 µL and 2000 mg/mL is placed in a 10 mL tube and 700 µL of the enzyme at 0.03 units/mL was placed on it and left for 10 min. An amount of 700 µL of the substrate was added to the mixture and incubated for 20 min. At the end of the period, the solution mixture was filtered with a 0.5 µm syringe filter and the absorbance value at 405 nm was recorded on the UV–vis spectrophotometer. DI water was used as control.

Briefly, in the TFC test, 0.1 mL of sample solution was placed in 10 mL glass tubes, and a 1.25 mL of FC solution was added, followed by the addition of 1 mL of saturated bicarbonate solution. At the end of 2 h, the mixed solution was filtered, and the absorbance value was recorded at 760 nm with a UV–vis spectrophotometer. Results were calculated based on gallic acid (GA) equivalent.

For the TFC test, 0.5 mL of sample solutions were prepared and then diluted by adding 2 mL of DI water. This medium was then treated with 0.15 mL of 5% NaNO_2_ and then 0.15 mL of 10% AlCl_3_·6H_2_O was added to this medium. Subsequently, 1 M 1 mL NaOH was added to the mixture. The UV–Vis spectrum of this solution was recorded at 405 nm after it was kept at rest for an additional 15 min. Rosmarinic acid (RA) was used as a standard.

#### 2.4.3. Antibacterial Activity Assay for PDA Coated g-C_3_N_4_

The antimicrobial activity of 20 mg/mL of g-C_3_N_4_-based materials was tested against Gram-negative *E. coli* (ATCC 8739) and Gram-positive *S. aureus* (ATCC 6538) for 24 h of incubation time. The g-C_3_N_4_, 1PDA@g-C_3_N_4_, and 3PDA@g-C_3_N_4_ were protonated for 1 h at room temperature interacting with 20 mL of 1 M HCl solutions. To remove excess HCl from the structure, the protonated PDA-5th@CNTs were centrifuged at 10,000 rpm and washed twice with DI water. The protonated and washed g-C_3_N_4_-based materials were dried using a freeze dryer as mentioned above. Microdilution tests were applied to the g-C_3_N_4_, 1PDA@g-C_3_N_4_, 3PDA@g-C_3_N_4_, and their protonated forms at a 20 mg/mL concentration. The suspensions were prepared in 10 mL of nutrient broth after sterilization and inoculated with 0.1 mL of bacteria suspension at 10^8^ CFU/mL adjusted to McFarland No: 0.5. A shaker was used to cultivate the suspension at 35 °C for 24 h. The suspensions were then inoculated on nutrient agar at temperatures of 35 °C for 24 h. After incubation, the colonies were counted, and the viability of the bacteria was determined.

The blood compatibility of the prepared PDA@g-C_3_N_4_ was determined by hemolysis and blood coagulation tests in accordance with the procedures available in the literature [32]. Assay details for hemolysis and blood clotting are provided in the Supplementary Material.

## 3. Results

**Figure 1:**
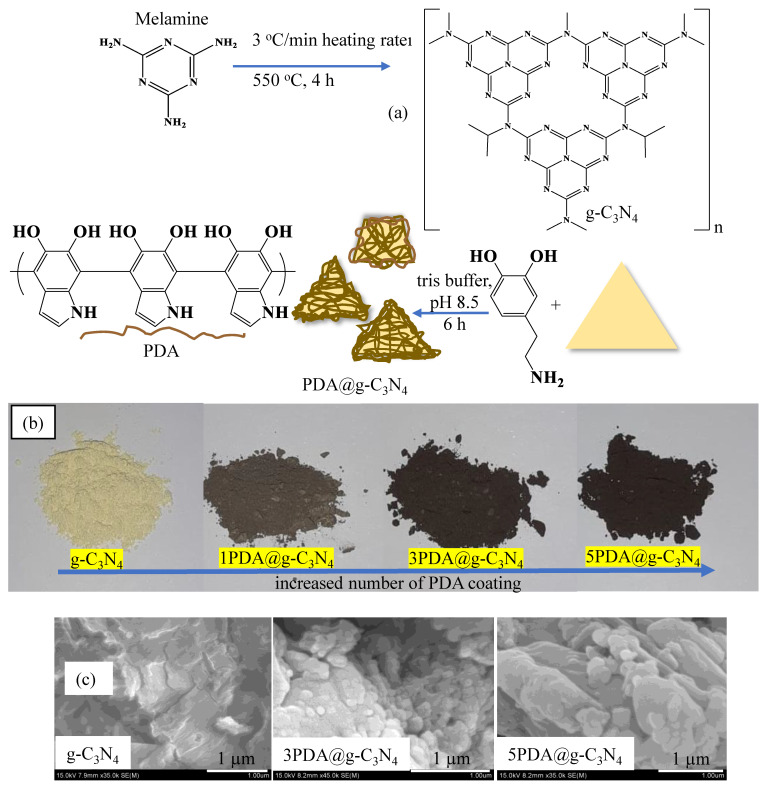
(**a**) Schematic representation of synthesis and PDA coating of g-C_3_N_4_, and (**b**) digital camera images of g-C_3_N_4_ and PDA@g-C_3_N_4_s, and (**c**) SEM images of g-C_3_N_4_ and PDA-coated g-C_3_N_4,_ 3PDA@g-C_3_N_4_ and 3PDA@g-C_3_N_4_.

Graphitic nitride (g-C_3_N_4_) has a layered assembly of corresponding plates that is similar to graphite [33,34]. It is reported that the carbon–nitrogen (C–N) bonds within g-C_3_N_4_ structures can be constructed from various sources including urea, dicyandiamide, melamine, etc. [27,28], and therefore they are in s-triazine and heptazine [35,36,37] structures. The schematic presentation of the prepared g-C_3_N_4_ structure is illustrated in Figure 1a. Surface functionalization of graphitic materials is an important route for creating active sites to anchor as well as to tune the materials’ chemical, electrical, optical, and even biological properties [38,39]. Many techniques have been successfully employed to construct advanced carbon-based functional materials to exploit these feature for diverse applications [40]. Among the modification methods employed in g-C_3_N_4_ structures, covalent and noncovalent modification techniques are the most often utilized [40]. The covalent modification procedures are not favored due to the material’s sp^2^ structure being damaged and the process being difficult [40].

Noncovalent techniques, on the other hand, offer easier application processes and have no negative impact on the original structure [40]. In contrast to other approaches, surface modification via PDA coating can offer a unique ability to deposit a thin film of a biocompatible polymer onto organic and inorganic surfaces of fundamentally any shape and size of materials [41,42,43]. In this study, PDA coating was employed as the surface modification technique and applied on g-C_3_N_4_, and the related schematic representation is given in Figure 1a. Accordingly, the g-C_3_N_4_ structures at a concentration of 2 mg/mL were placed in the DA solution with 500 mL of 10 mM pH 8.5 tris buffer solution which afforded PDA coating. PDA possesses highly expressed catechol and amino functions, which enable this polymer to coat almost any substrate with a variety of forms and geometries by emulating the structure of the adhesive protein found in mussels [44,45]. The coating process with PDA was performed five times consecutively on g-C_3_N_4_, and the first, third, and fifth coatings as 1PDA@g-C_3_N_4_, 3PDA@g-C_3_N_4_, and 5PDA@g-C_3_N_4_ were tested for the application of biomedical parameters. The change in color of g-C_3_N_4_ from clear yellowish to black with an increasing number of PDA coatings is shown in digital camera images in Figure 1b. The changes in the intensity of the dark coloration of g-C_3_N_4_ with the increasing number of PDA coatings is clearly visible in the digital camera images as illustrated in Figure 1b. The increase in the number of coatings provides a higher PDA amount to the surface of the g-C_3_N_4_ structures as revealed with digital camera images. From the SEM images in Figure 1c, the morphology of g-C_3_N_4_ after coating with PDA did not significantly change regarding the size of the flakes. However, some PDA-coated g-C_3_N_4_ in nano, submicron, and micrometer sizes are visible on the surfaces.

**Figure 2:**
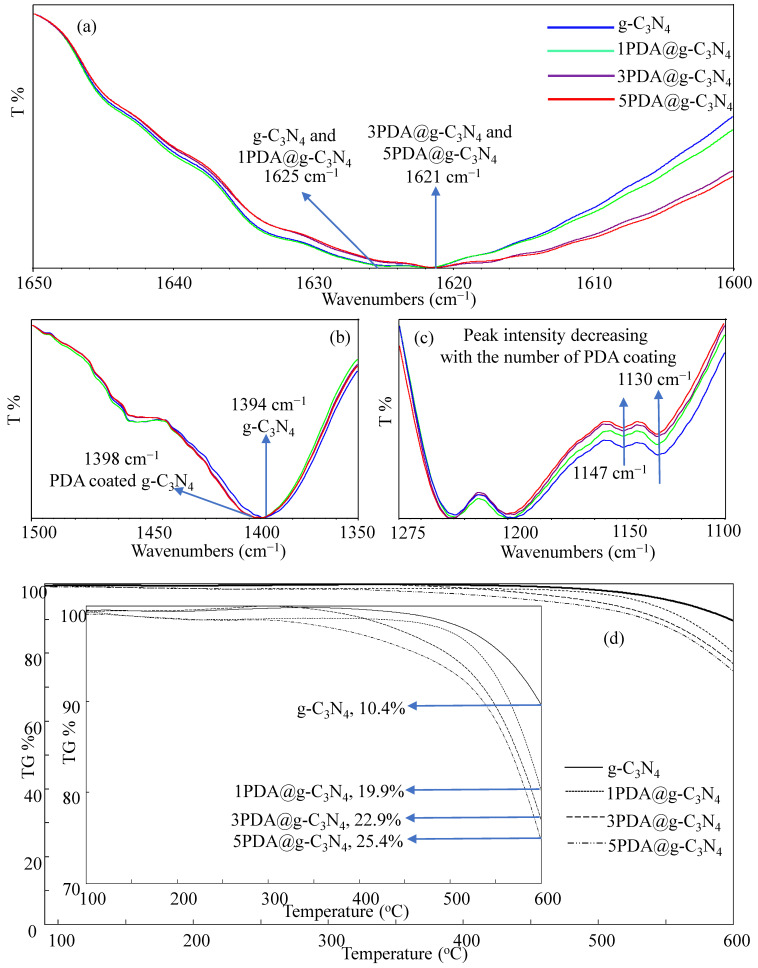
Comparison of FT-IR spectrum in details between (**a**) 1600–1650 cm^−1^, (**b**) 1350–1500 cm^−1^, (**c**) 1100–1275 cm^−1^, and (**d**) TGA thermograms of g-C_3_N_4_ and PDA@g-C_3_N_4_ structures.

The FT-IR spectra of the g-C_3_N_4_ and PDA@g-C_3_N_4_ structures are compared and presented in Figure S1. As can be seen, there is no noticeable change visible. To better assess the PDA coatings on g-C_3_N_4_ surfaces, the FT-IR spectra of the g-C_3_N_4_ and PDA-coated 1PDA@g-C_3_N_4_, 3PDA@g-C_3_N_4_, and 5PDA@g-C_3_N_4_ structures are compared in detail and given in in Figure 2a–c, respectively. Due to the normal stretching modes of aromatic C-N heterocyclic structures, the bands in the 1200–1600 cm^−1^ region were seen in all spectra. Triazine unit vibrations are present at 807 cm^−1^ for all g-C_3_N_4_-based materials [46,47]. At 3000–3500 cm^−1^, the peaks corresponding to the vibrations of terminal amino groups and surface-adsorbed –OH bands are visible also for all g-C_3_N_4_-based materials [48,49]. However, due to overlapping bands in the FT-IR spectra of both g-C_3_N_4_s and PDAs, no substantial change in the FT-IR spectra of the g-C_3_N_4_ and PDA@g-C_3_N_4_ structures was observed. Therefore, the detailed FT-IR spectra of the three regions, e.g., 1600–1650 cm^−1^, 1350–1500 cm^−1^, and 1100–1275 cm^−1^, are shown in Figure 2a–c, respectively. It is also apparent that there are not very significant changes in the FT-IR spectrum of these regions, as the N–H stretching and indole ring stretching peaks which are the most likely characteristic peaks to be observed for PDA at 1605 and 1514 cm^−1^, respectively, cannot be seen due to the overlapping of the strong peak belonging to g-C_3_N_4_ in this region [26]. However, there is a slight peak shift upon PDA coating that is observed in Figure 2a, e.g., the 1625 cm^−1^ peak belonging to g-C_3_N_4_ shifted to 1621 cm^−1^ upon PDA coating. On the other hand, the observed peak at 1394 cm^−1^ in the FT-IR spectrum of g-C_3_N_4_ shown in Figure 2b shifted to 1398 cm^−1^ after the PDA coating process. Also, there is some decrease in intensities of the peaks at 1147 and 1130 cm^−1^ upon PDA coating as illustrated in Figure 2c.

Because the PDA coating of the g-C_3_N_4_ structures cannot be adequately confirmed by FT-IR spectra, TGA thermograms of the uncoated g-C_3_N_4_ and PDA@g-C_3_N_4_ structures were compared to evaluate and compare the thermal stability of the structures and to validate the increasing quantity of PDA coating on g-C_3_N_4_ with the repeated coating procedures, and the corresponding thermograms are shown in Figure 2d. Because the synthesis of the g-C_3_N_4_ structures is performed at 550 °C, the uncoated g-C_3_N_4_’s thermal degradation began after around 550 °C. At 600 °C, it was observed that the g-C_3_N_4_ structures lost 10.6% of their mass. On the other hand, the PDA@g-C_3_N_4_ structures had essentially little mass loss up to 550 °C. However, at 600 °C, the thermal degradation of the 1PDA@g-C_3_N_4_, 3PDA@g-C_3_N_4_, and 5PDA@g-C_3_N_4_ structures was 19.9%, 22.9%, and 25.4%, respectively. This is due to an increase in the quantity of PDA coating on the g-C_3_N_4_ structures as the number of PDA coatings increases. The quantity of PDA in the structure after the first coating was determined to be 9.3%, and after the third and fifth coatings, the amount of PDA on the g-C_3_N_4_ structures grew to 12.3% and 14.8%, respectively.

For detailed information to confirm the increase in PDA amount on the g-C_3_N_4_ structures, the EDX analysis of the uncoated g-C_3_N_4_, 1PDA@g-C_3_N_4_, 3PDA@g-C_3_N_4_, and 5PDA@g-C_3_N_4_ structures were carried out, and corresponding results are summarized in Table 1. It was clearly seen that the elemental ingredients of uncoated g-C_3_N_4_ includes 22.1% C, 71.8% N, and 5.4% O. The amount of O in neat g-C_3_N_4_ is 5.4 wt% which comes from the air as the g-C_3_N_4_ was synthesized in air atmosphere. So, the amount of PDA coatings of g-C_3_N_4_ was determined as 4.4%, 7.7%, and 14.4% weight increases for 1PDA@g-C_3_N_4_, 3PDA@g-C_3_N_4_, and 5PDA@g-C_3_N_4_ that are normalized based on the amount of O atoms in the neat g-C_3_N_4_.

On the other hand, from the EDX analysis of the PDA-coated g-C_3_N_4_ structure, it can be clearly seen that the percentage of C and O atoms increased as the number of PDA coatings increased, while the percentage of N atoms decreased. The determined C% for the structures 1PDA@g-C_3_N_4_, 3PDA@g-C_3_N_4_, and 5PDA@g-C_3_N_4_ are 23.2%, 26.0%, and 32.6%, respectively. On the other hand, %N for the 1PDA@g-C_3_N_4_, 3PDA@g-C_3_N_4_, and 5PDA@g-C_3_N_4_ structures were calculated as 67.0, 60.9, and 48.3%, respectively. This is because for every PDA unit, there are 8 C atoms generated versus 1 N atom and 2 O atoms formed. As the number of PDA coatings increases, the amount of O which increases on the coated g-C_3_N_4_ reveals coatings of PDA is accomplished. As observed for O atoms, there are 9.8, 13.1, and 19.1 wt% for the first, third, and fifth PDA coatings on g-C_3_N_4_, versus 5.4 wt% for uncoated g-C_3_N_4_.

**Figure 3:**
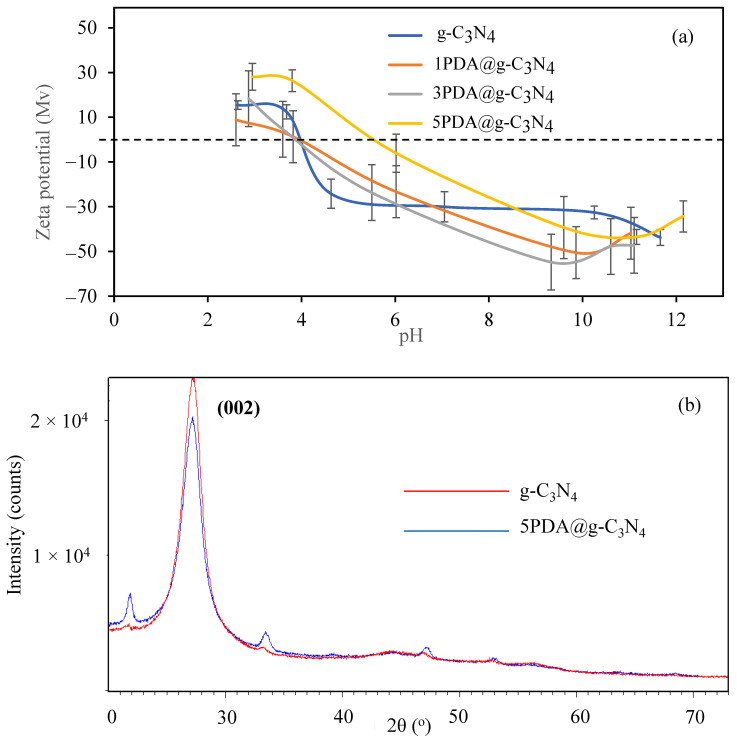
Comparison of (**a**) pH–zeta potential graphs, and (**b**) XRD patterns of g-C_3_N_4_ and PDA@g-C_3_N_4_ structures.

Furthermore, the changes in the zeta potential of the g-C_3_N_4_ and PDA@g-C_3_N_4_ structures at various pHs of the particle solution was investigated and the results are given in Figure 3a. The zeta potential values for g-C_3_N_4_, 1PDA@g-C_3_N_4_, 3PDA@g-C_3_N_4_, and 5PDA@g-C_3_N_4_ were calculated from their dispersion in 1 mM KNO_3_ solution at 1 mg/mL concentration, and were measured as −23.3 ± 3.0, −30.0 ± 6.8, −23.7 ± 12.6, and −6.1 ± 8.5 mV, respectively. The isoelectric points of the g-C_3_N_4_, 1PDA@g-C_3_N_4_, 3PDA@g-C_3_N_4_, and 5PDA@g-C_3_N_4_ structures were determined as pH 4.0, 4.0, 3.9, and 5.6, respectively. The zeta potential values of the structures are zero at these pH values, whereas they are positively charged at pH values lower than these values, and they become negatively charged at higher pH values. In addition, to ascertain the effect of KNO_3_ concentration on the zeta potential of the g-C_3_N_4_ and PDA@g-C_3_N_4_ structures, the measurements were conducted at various concentrations of KNO_3_ solution. As seen in Figure S2, the zeta potential values of g-C_3_N_4_ and PDA@g-C_3_N_4_ were not significantly affected by KNO_3_ solution concentrations ranging from 1 to 100 mM. However, when KNO_3_ solution is not used as the measuring medium, big jumps in pH values occur immediately upon small additions of acid or base in the medium during the adjustment of solution pHs, resulting in incorrect measurements. The ionic strength of the medium needs to be equilibrated by use of some salts, e.g., KNO_3_, as some materials that can develop charges upon small additions of acid or base used to arrange pH of the medium can influence the medium pH greatly. Therefore, zeta potential measurements were generally performed in salt solutions with low concentrations such as 1–100 mM [27].

Moreover, the XRD pattern comparison of the g-C_3_N_4_ and 5PDA@g-C_3_N_4_ structures are also given in Figure 3b. An XRD peak associated with in-plane structural packing is easily recognized from the exact periodic units in each layer of g-C_3_N_4_. The typical experimental XRD pattern of bulk g-C_3_N_4_ contains a unique diffraction peak positioned at 2θ = 27.40°, which is indexed as graphitic materials’ (002) diffraction plane. In the literature, two main peaks, 2θ = 13.40 and 2θ = 27.40 for g-C_3_N_4_ at (100) and (002) planes, are reported [50,51]. The first peak is the structural packing between the layers and the second peak is the characteristic interplanar staking peaks of the aromatic systems. As the 2θ range of 20°–73° is shown in Figure 3b, the peak at 27° is clearly visible. It can also be seen that the peak intensity decreases slightly due to the dopamine coating, e.g., the intensity of 5PDA@g-C_3_N_4_ is 20,315 at 2θ = 27°, whereas the intensity of g-C_3_N_4_ is 23,129 at the same 2θ value.

According to the XRD data, the g-C_3_N_4_ has a flake-like structure with an interplanar stacking distance of 0.356 nm as shown by (002) diffraction. However, there is also no significant changes in the structure of g-C_3_N_4_ after PDA coating, and the diffraction peak was observed at 27.4° 2θ, indicating that the material is still in a graphitic structure with reduced intensity as an indication of the PDA coating [49].

**Figure 4:**
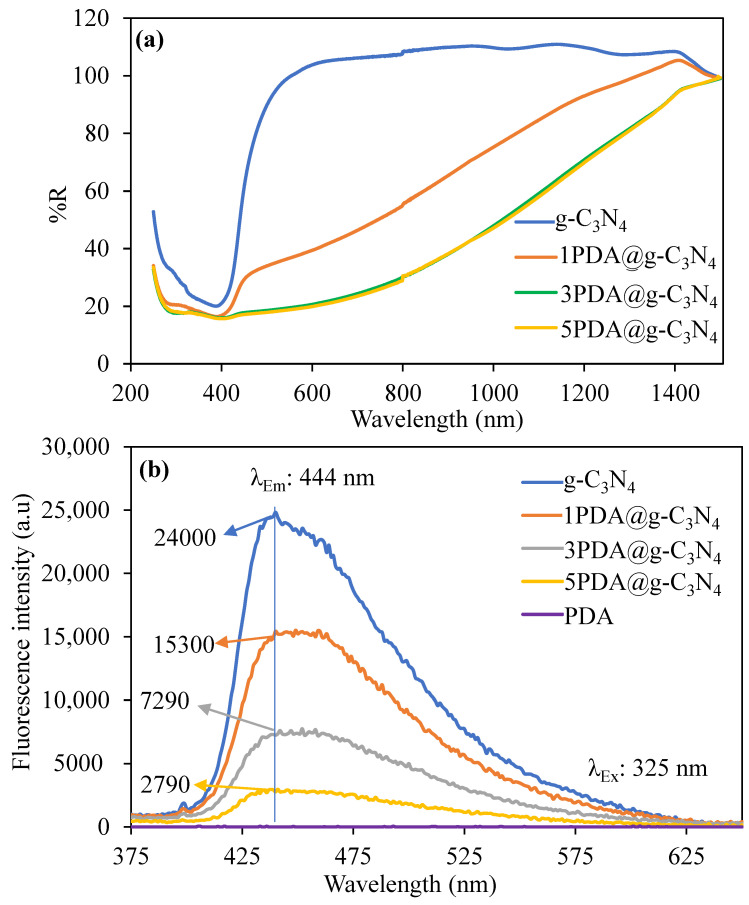
Comparison of (**a**) reflectance spectrums, and (**b**) fluorescence spectra of g-C_3_N_4_ and PDA@g-C_3_N_4_ structures.

The change in optical properties of g-C_3_N_4_ after multiple PDA coatings was also investigated using bandgap analysis and the change in fluorescence properties of the structures. Figure 4a depicts the UV–Vis DRS spectra of the g-C_3_N_4_ and PDA@g-C_3_N_4_ structures, and Figure S3 shows the reflectivity wavelength diagram used to determine the band gaps of the g-C_3_N_4_-based structures. The band gap of g-C_3_N_4_ is determined to be 2.72 eV, which is consistent with previously reported g-C_3_N_4_ structures [31,52,53,54]. The coating of g-C_3_N_4_ with PDA, on the other hand, shifts the band gap to lower levels. The bandgap values for 1PDA@g-C_3_N_4_ after the first PDA coating were determined to be 2.57 and 0.94 eV. Because of non-homogeneous coating, the first PDA coating results in two band gap values one of which is for g-C_3_N_4_ with a value of 2.57 eV that is closer to bare g-C_3_N_4_ and another band gap with a value of 0.94 eV for incomplete PDA coating or partial coating of g-C_3_N_4_. However, following the third and fifth PDA coatings of g-C_3_N_4_, single band gap values were measured as 0.89 and 0.87 eV, respectively.

The UV–Vis spectra of neat g-C_3_N_4_ and the PDA-coated g-C_3_N_4_ structures at 0.2 mg/mL concentrations based on g-C_3_N_4_ amounts are given in Figure S4a. Based on EDX results provided in Table 1, the amount of PDA coating of the g-C_3_N_4_s structures (normalized according to the O) are calculated as 4.4%, 7.7%, and 14.4% after the frist, third, and fifth coating processes, respectively. To eliminate the concentration dependence of absorbance values, the aqueous solutions of g-C_3_N_4_, 1PDA@g-C_3_N_4_, 3PDA@g-C_3_N_4_, and 5PDA@g-C_3_N_4_ weighing 20, 20.9, 21.5, and 22.9 mg, respectively, per 1 mL water solution were prepared and diluted 1000-fold for the comparison of their corresponding UV–Vis spectra, shown in Figure S4a. Thus, each sample, g-C_3_N_4_, 1PDA@g-C_3_N_4_, 3PDA@g-C_3_N_4_, and 5PDA@g-C_3_N_4_, have 0.0200 mg g-C_3_N_4_ per 1 mL solution. As can be seen in Figure S4a, the max absorbance values for the g-C_3_N_4_-based structures were observed at 325 nm and are 0.086, 0.082, 0.069, and 0.053 for g-C_3_N_4_, 1PDA@g-C_3_N_4_, 3PDA@g-C_3_N_4_, and 5PDA@g-C_3_N_4_, respectively. So, the decrease in absorbance values at 325 nm of these g-C_3_N_4_ structures with an increasing number of PDA coatings directly related to the increased amount of PDA on the surface of g-C_3_N_4_s. The digital camera images shown in Figure S4b contain the same amounts of g-C_3_N_4_s, 0.02 mg/mL.

Accordingly, the excitation wavelengths of the g-C_3_N_4_, PDA-coated g-C_3_N_4_ structures, and PDA were determined at 325 nm, and their emission wavelengths and fluorescence intensities were compared in the 350–600 nm range. The fluorescence emission spectra of g-C_3_N_4_ the PDA-coated g-C_3_N_4_ structures, 1PDA@g-C_3_N_4_, 3PDA@g-C_3_N_4_, and 5PDA@g-C_3_N_4_, and PDA are given in Figure 4b. The emission wavelength for all structures was determined at 444 nm, but differences were observed in the fluorescence emission intensities, except for PDA. PDA did not show any fluorescence properties at this excitation wavelength. The structures were excited at 325 nm, which corresponds to the S_0_-to-S_1_ transition, and gave off fluorescence emissions around 450 nm, showing that the S_1_-to-S_0_ transition occurred [55]. Accordingly, while the observed fluorescence emission intensity for the g-C_3_N_4_ structure at 444 nm wavelength is 24,000, this value decreases to 15,300, 7290, and 2790 for the 1PDA@g-C_3_N_4_, 3PDA@g-C_3_N_4_, and 5PDA@g-C_3_N_4_ structures, respectively. As expected, there was a decrease in the fluorescence properties of the g-C_3_N_4_ structures after coating with PDA and decrease in intensity of the fluorescence properties increased as the number of coatings with PDA increases. Although the concentrations of the solutions used were the same (0.02 mg/mL based on g-C_3_N_4_) in the measurements with florescence spectroscopy, the resulting decrease in fluorescence intensity with number of PDA coatings is due to the suppression of the fluorescence feature coming from the g-C_3_N_4_ structure. Table 2 also summarizes the QY% values calculated based on Equation (2) for g-C_3_N_4_, 1PDA@g-C_3_N_4_, 3PDA@g-C_3_N_4_, and 5PDA@g-C_3_N_4_.

The QY% values of the materials with luminous characteristics are important in their optical use. The higher the fluorescence property, the higher the QY%. As given in Table 2, the QY% value of g-C_3_N_4_ is 19.8 ± 1.2, with 13.7 ± 1.1, 9.1 ± 0.7, and 4.8 ± 0.6% calculated for the 1PDA@g-C_3_N_4_, 3PDA@g-C_3_N_4_, and 5PDA@g-C_3_N_4_ structures, respectively. As expected, with the increase in number of PDA coatings of g-C_3_N_4_, the QY values decreased due to the increased extent of interaction of the excited electrons with the increased number PDA chains with a higher number of coatings.

**Figure 5:**
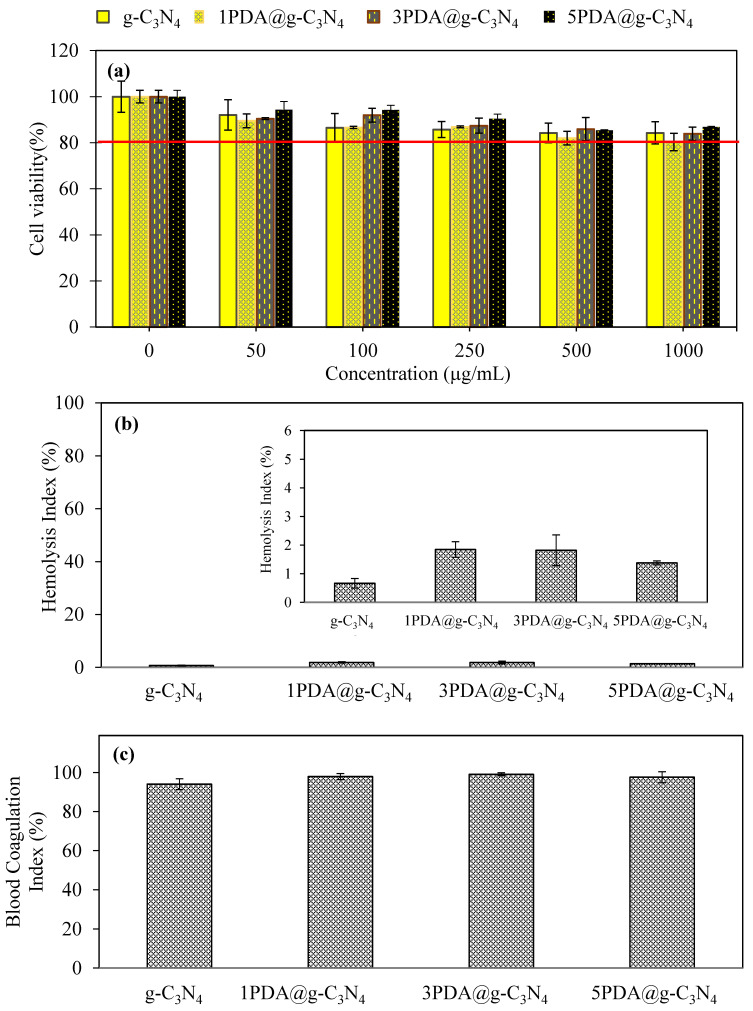
(**a**) Biocompatibility against fibroblast cells, (**b**) hemolysis and (**c**) blood clotting index of g-C_3_N_4_ and PDA@g-C_3_N_4_ structures.

Cytotoxicity tests of g-C_3_N_4_ and PDA@g-C_3_N_4_ were performed on L-929 fibroblast cells and the results are shown in Figure 5. Cell viability of g-C_3_N_4_ was found >80% in all concentrations in the range of 50–1000 ug/mL.

The biocompatibility results confirm that g-C_3_N_4_ is considered biocompatible up to a concentration of 1000 µg/mL. Polydopamine coating on g-C_3_N_4_ did not affect its biocompatibility, as cell viability results showed 81.9 ± 4.8% cell bioavailability for g-C_3_N_4_ for 1000 µg/mL concentration versus cell viabilities of 80.3 ± 3.8, 83.9 ± 1.0 and 86.8 ± 1.0% for the first, third, and fifth PDA-coated g-C_3_N_4_, respectively.

The hemolysis and blood clotting index values of g-C_3_N_4_ and PDA@g-C_3_N_4_ are illustrated in Figure 5b and 5c, respectively. Hemolysis index values were determined as 0.7 ± 0.2% for g-C_3_N_4_, whereas it was determined as 1.8 ± 0.5% for 3PDA@g-C_3_N_4_ which was the highest among all the PDA-coated g-C_3_N_4_s. As the hemolysis% values for all the samples are <5%, they are all blood compatible. Or in other words, g-C_3_N_4_ materials whether PDA-coated or not and independent of the number of PDA coatings, do not rupture red blood samples and can be utilized in blood contacting applications safely. Another important parameter in the blood compatibility test is the blood clotting index% which gives information on whether the material in question influences the clotting mechanism of the blood. In order not to impair the clotting mechanism of the blood, the blood clotting percentage of a material must be around 100% [56]. As shown in Figure 5c, the lowest blood clotting index value was determined for g-C_3_N_4_ with 94.1 ± 2.8%, and PDA-coated g-C_3_N_4_, 1PDA@g-C_3_N_4_, 3PDA@g-C_3_N_4_, and 5PDA@g-C_3_N_4_ demonstrated the blood clotting% values of 97.92 ± 1.2, 99.1 ± 0.8%, and 97.62 ± 1.5%, respectively, ascertaining that PDA coating improves the blood compatibility of g-C_3_N_4_ by not interfering with the mechanism of the clotting of blood.

**Figure 6:**
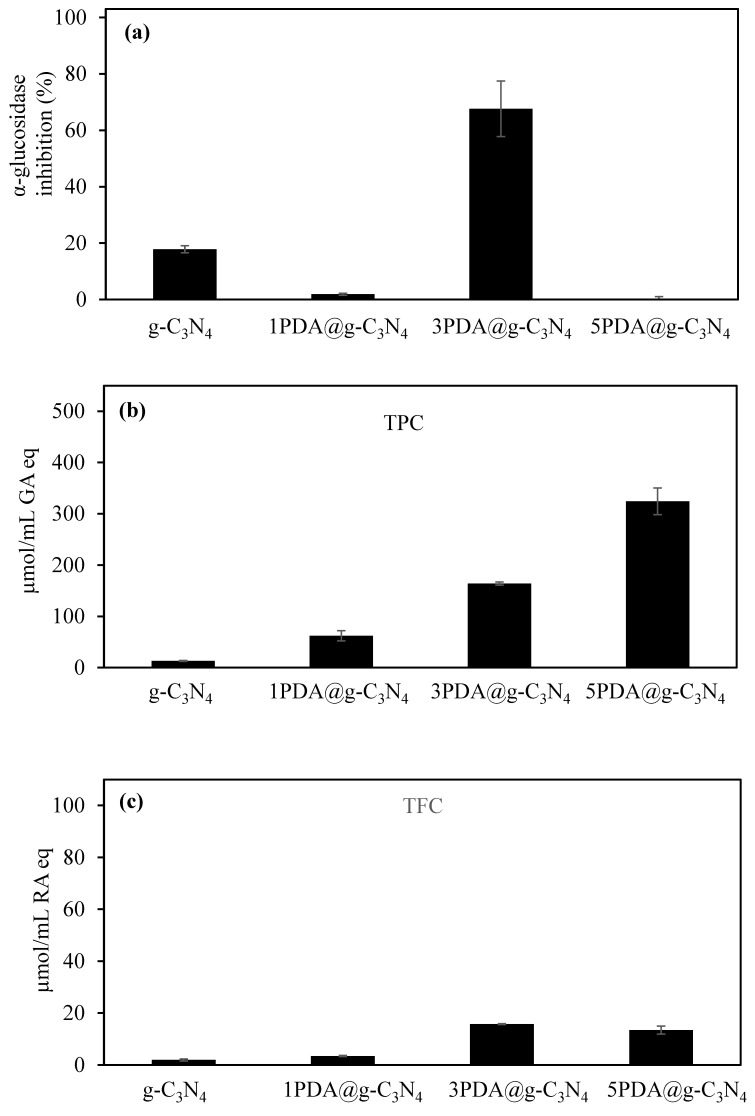
(**a**) α-Glucosidase inhibition, (**b**) total phenol content (TPC), and (**c**) total flavonoid content (TFC) of g-C_3_N_4_ and PDA@g-C_3_N_4_ structures.

In addition, the treatment of type II diabetes also considers other aspects that are important for biomedical application, such as the inhibition of enzymes, e.g., α-glucosidase, a hydrolysis enzyme that breaks down disaccharides and α-glucosidase inhibitors. Therefore, the α-glucosidase enzyme inhibitory ability of g-C_3_N_4_ and its PDA-coated forms was investigated, and the results are summarized in Figure 6a. As can be seen amongst all the g-C_3_N_4_-based materials, the highest α-glucosidase inhibition potency was determined for 3PDA@g-C_3_N_4_ with 67.6 ± 9.8%, suggesting the potential use of PDA-coated materials in type II diabetes.

Furthermore, the determine the antioxidant effectiveness of PDA-coated g-C_3_N_4_, the total phenol content (TPC) and total flavonoid content of (TFC) test were performed at 2000 µg/mL for g-C_3_N_4_ and PDA@g-C_3_N_4_ samples and results are shown in Figure 6b and 6c, respectively. According to the TPC test results, there is linear increase in TPC values in parallel to the PDA coating number and the highest TPC value of 314 ± 26 µmol/mL GA eq was found for 5PDA@g-C_3_N_4_. On the other hand, TFC values for the PDA coating of g-C_3_N_4_ show a slight increase with the number of PDA coatings of g-C_3_N_4_, as shown in Figure 6c. In the TFC test, the highest value obtained was 15.7 ± 0.2 mg/mL RA eq for the 3PDA@g-C_3_N_4_ sample.

While the TPC of PDA-coated samples shows a linear increase with an increased number of coatings, suggesting antioxidant properties, the TFC value showed only a marginal increase with PDA coating; this is reasonable, as DA is a phenolic compound, not a flavonoid.

To evaluate the antibacterial capabilities of g-C_3_N_4_ and its PDA-coated forms, bacteria inhibition% values against two bacterial strains were examined using 20 mg/mL concentrations of g-C_3_N_4_ and PDA@g-C_3_N_4_, and their protonated form, PDA@g-C_3_N_4_^+^. As shown in Figure S5, g-C_3_N_4_ showed a reducing effect on the bacterial growth of *S. aureus* bacterium with reduction of 91.2 ± 0.4%. The highest inhibition% was observed as 62.3 ± 1.9% against *S. aureus*, and 76.5 ± 1.0% inhibition against *E. coli* for 3PDA@g-C_3_N_4_^+^. Overall, upon protonation all the g-C_3_N_4_ and PDA@g-C_3_N_4_ material showed increased inhibition% values against common pathogens, e.g., *S. aureus* and *E. coli*, indicating the potential role of PDA-coated g-C_3_N_4_ composites in preventive or prophylactic usage against infections caused by these microorganisms or protection against the spread and/or contamination of infections caused by different pathogens.

PDA is a versatile antibacterial agent and can be combined or modified with other materials or compounds to achieve a further potent antibacterial potency. These materials may include metal ions, antibiotics, and other antibacterial substances on the surface, resulting in controlled release, detoxification, or other effects. Therefore, PDA coating represents a simple and universal method for functionalizing various material surfaces as excellent antibacterial materials, making it a valuable addition to any material [57].

## 4. Conclusions

Here, we reported the facile multiple coating of g-C_3_N_4_ by PDA through self-polymerization of DA, a phenolic neurotransmitter. It was shown that the g-C_3_N_4_ 5PDA@g-C_3_N_4_, coated up to five times, retained the fluorescence properties, although the fluorescence emission at 444 nm was reduced by five times. As the coating number of the PDA increased, the intensity of fluorescence emission decreased linearly. It was found that three consecutive PDA coatings enabled complete coating with g-C_3_N_4_ and bestowed significant improvement in biomedical application potentials. For example, the cytotoxicity of the 1PDA@g-C_3_N_4_, 3PDA@g-C_3_N_4_, and 5PDA@g-C_3_N_4_ structures is retained against L929 and fibroblast cell lines and their blood compatibilities via hemolysis, and the blood clotting index% was enhanced in comparison to bare g-C_3_N_4_. Furthermore, the antioxidant capability of PDA-coated g-C_3_N_4_ via a total phenol content assay revealed a linear increased antioxidant ability in terms of GA equivalency with an increasing number of PDA coatings. Moreover, because of the presence of N atoms in g-C_3_N_4_ and the PDA structures, g-C_3_N_4_ and PDA@g-C_3_N_4_ can be easily protonated by simple acid treatments such as HCl, etc. Therefore, their protonated forms also resulted in a reduction in bacterial growth, further conforming the improved biomedical utilization of PDA-coated material. Due to the presence of a PDA coating on an optically active 2D g-C_3_N_4_, it is expected that this type of material could be used in the treatment of neurogenerative diseases such as Parkinson’s and Alzheimer’s.

## Figures and Tables

**Table 1:** EDX analysis results of g-C_3_N_4_-based structures.

Materials	Elements (wt%)		
	C	N	O
g-C_3_N_4_	22.1	71.8	5.4
1PDA@g-C_3_N_4_	23.2	67.0	9.8
3PDA@g-C_3_N_4_	26.0	60.9	13.1
5PDA@g-C_3_N_4_	32.6	48.3	19.1

**Table 2:** The quantum yield% (QY%) values of g-C_3_N_4_ and PDA@g-C_3_N_4_ structures.

Samples	g-C_3_N_4_	1PDA@g-C_3_N_4_	3PDA@g-C_3_N_4_	5PDA@g-C_3_N_4_
QY%	19.8 ± 1.2	13.7 ± 1.1	9.1 ± 0.7	4.8 ± 0.6

## Data Availability

Data are contained within the article and Supplementary Materials.

## Supplementary Materials

The following supporting information can be downloaded at: https://www.mdpi.com/article/10.3390/biomedicines12061151/s1, Figure S1: FT-IR spectrum of g-C_3_N_4_ and PDA-coated g-C_3_N_4_ structures. Figure S2: (a) pH–zeta potential plots of g-C_3_N_4_ and (b) 5PDA@g-C_3_N_4_ in different concentrations of KNO_3_ solutions (0, 1, 10, 100 mM). Figure S3: The calculated bandgap values for (a) g-C_3_N_4_, (b) 1PDA@g-C_3_N_4_, (c) 3PDA@g-C_3_N_4_, and (d) 5PDA@g-C_3_N_4_. Figure S4: (a) UV–Vis spectra and (b) digital camera images of g-C_3_N_4_ and PDA-coated g-C_3_N_4_ structure solutions at 0.2 mg/mL concentration of g-C_3_N_4_s. Figure S5: Antimicrobial activity of 20 mg/mL of g-C_3_N_4_-based materials against Gram-negative *E. coli* (ATCC 8739) and Gram-positive *S. aureus* (ATCC 6538) for 24 h of incubation time.
