# Supplementary material for: Polydopamine Coating of Graphitic Carbon Nitride, g-C3N4, Improves Biomedical Application
Source: Biomedicines. 2024 May 23;12(6):1151. doi: 10.3390/biomedicines12061151 (PMC11201011; doi:10.3390/biomedicines12061151)
Supplement: Supplementary file 1 [file biomedicines-12-01151-s001.zip › biomedicines-2919093-SI.pdf]

Supplementary Materials

Polydopamine Coating of Graphitic Carbon Nitride, g-C_3N_4 Improves Biomedical Application

Mehtap Sahiner [1], Sahin Demirci [2] and Nurettin Sahiner [2,3]*

[1] Department of Bioengineering, Faculty of Engineering, Canakkale Onsekiz Mart University Terzioglu Campus, 17100 Canakkale, Turkey
[2] Department of Chemistry, Faculty of Sciences, Canakkale Onsekiz Mart University Terzioglu Campus, 17100 Canakkale, Turkey
[3] Department of Ophthalmology, Morsani College of Medicine, University of South Florida, 12901 Bruce B Downs B. Downs Blv., MDC 21, Tampa, FL 33612, USA
* Correspondence: E-mail: sahiner71@gmail.com or nsahiner@usf.edu; Tel.: +1-813-974-0135; Fax: +1-813-974-5621

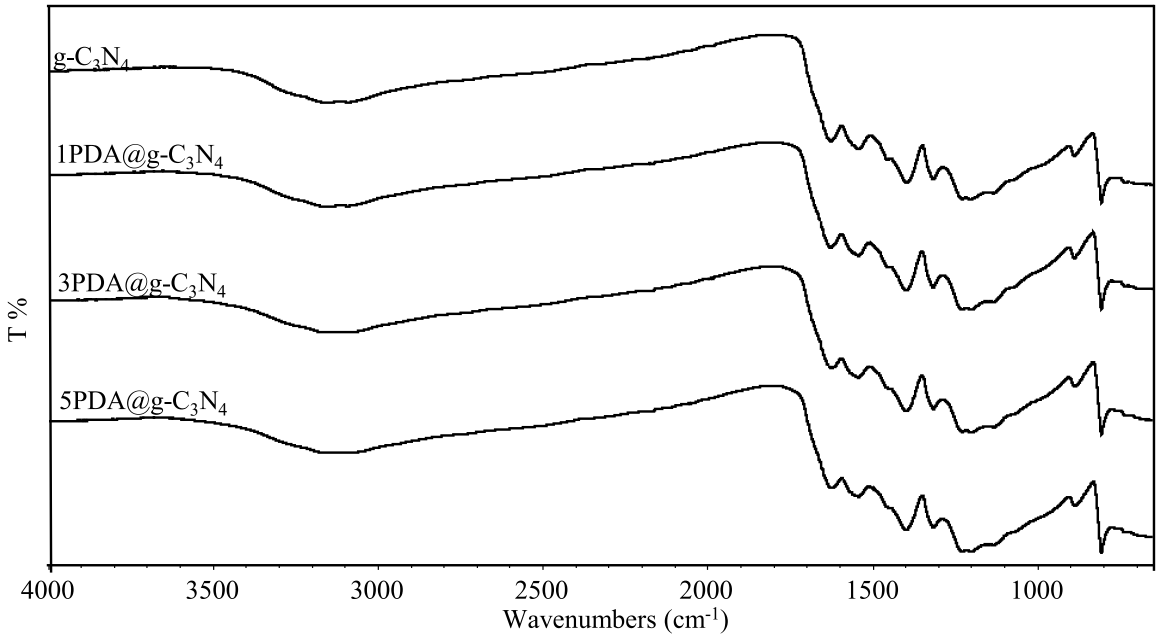

Figure S1. FT-IR spectrum of $g\text{-}C_3N_4$ and PDA coated $g\text{-}C_3N_4$ structures.

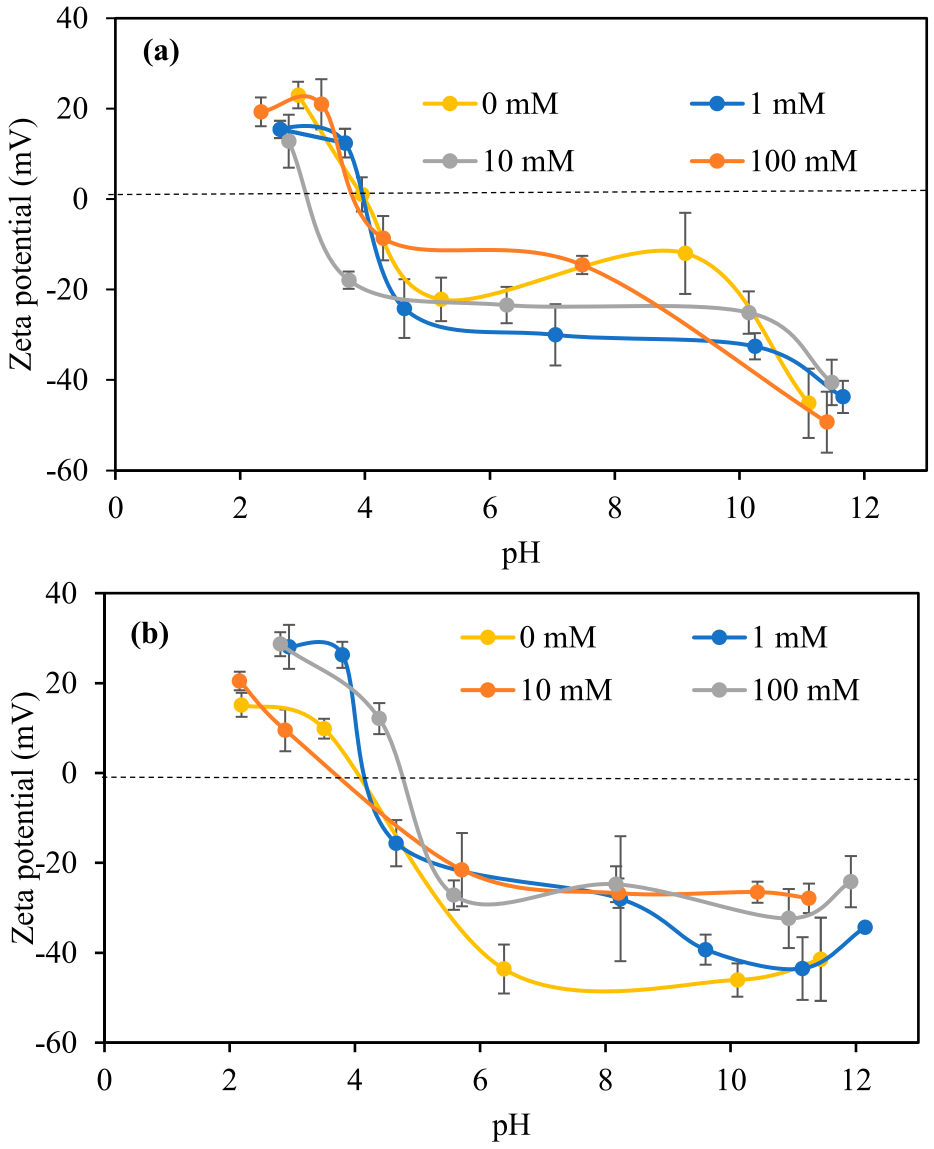

Figure S2. (a) pH-zeta potential plots of g-C_3N_4 and **(b)** 5PDA@g-C_3N_4 in different concentrations of KNO_3 solutions (0, 1, 10,100 mM).

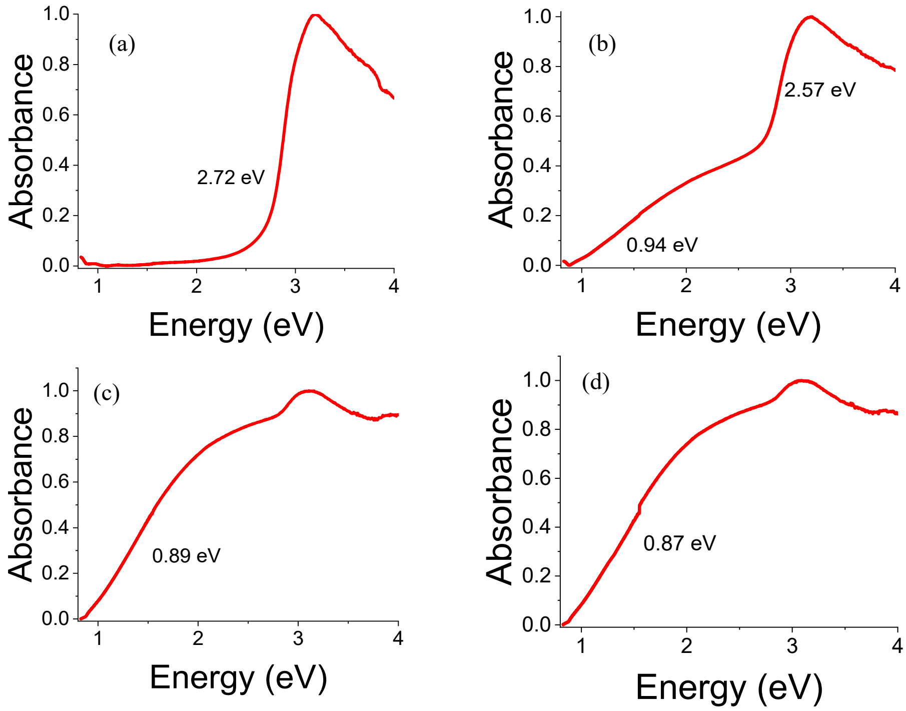

Figure S3. The calculated bandgap values for (**a**) g-C_3N_4, (**b**) 1PDA@g-C_3N_4, (**c**) 3PDA@g-C_3N_4, and (**d**) 5PDA@g-C_3N_4.

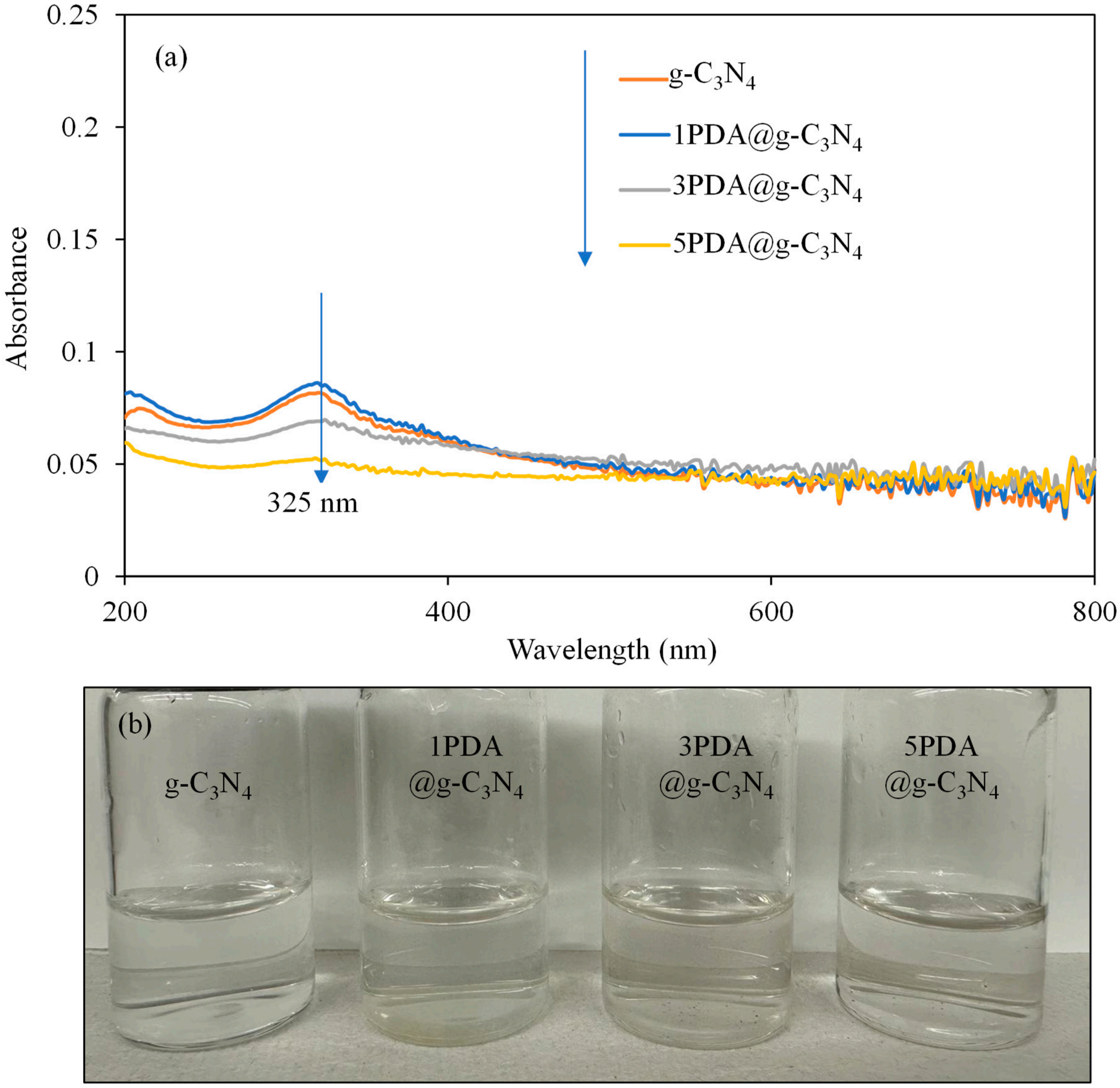

Figure S4. (**a**) UV-Vis spectra and (**b**) digital camera images of g-C_3N_4 and PDA coated g-C_3N_4 structure (each containin 0.02 mg/mL g-C_3N_4).

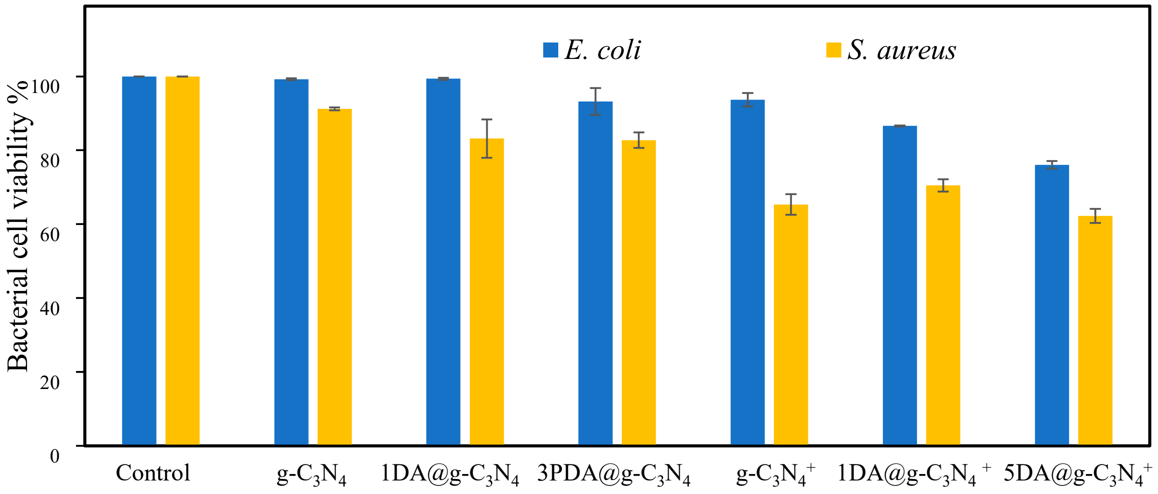

Figure S5. Antimicrobial activity of 20 mg/mL of g-C3N4-based materials against Gram-negative *E. coli* (ATCC 8739) and Gram-positive *S. aureus* (ATCC 6538) for 24 h incubation time.

Hemolysis test

For the hemocompatibility tests, Human whole blood was taken from healthy volunteers. The fresh blood was put into hemogram tubes containing EDTA and slowly shaken.

To conduct a haemolytic study, a solution of 2D materials based on g-C_3N_4 was prepared in 0.9% saline solution at a concentration of 1 mg/mL. The solution was then kept at a temperature of 37.5 °C. Anticoagulant mixed with 2 mL of blood was diluted with 2.5 mL of 0.9% saline solution. Next, 0.2 mL of the diluted blood solution was added to 10 mL of the g-C_3N_4 suspension, which was then incubated for 1 hour at 37.5 ºC in a shaker bath. After incubation, the suspensions were centrifuged at 100g for 5 minutes. The released amount of haemoglobin was determined by measuring the absorbance of the supernatant solutions using a UV–vis spectrophotometer at 542 nm. The hemolysis % ratio was calculated from Eq. (S1):

$$\text{Hemolysis ratio } \% = (A_{sample} - A_{negative})/(A_{positive} - A_{negative}) \times 100 \quad \text{(S1)}$$

To determine the absorbance of the sample-containing blood solution, 0.2 mL of diluted blood was mixed with 10 mL of DI water, and 0.2 mL of diluted blood was mixed with 10 mL of saline solution. These samples were labeled as A_{Sample}, $A_{positive}$, and $A_{negative}$, respectively. The hemolysis tests were conducted in triplicates, and the data is presented as the average of these values along with the standard deviations.

For the blood clotting study, 3 mL of anticoagulant-containing blood was mixed with 0.24 mL of 0.2 M $CaCl_2$ solution, and the mixture was kept agitated slowly. Then, 0.27 mL of this blood solution was dropped onto 10 mg of GA-based microgels in flat-bottomed tubes until the sample was completely covered. The medium was then incubated in a water shaker bath at 37.5 °C for 10 minutes. After the incubation, 10 mL of DI water was slowly added to the medium, and the mixture was immediately centrifuged at 100g for 30 seconds. The supernatant solution was taken, and it was diluted with 40 mL of DI water. This blood medium was then incubated in a shaking bath at 37.5 °C for 1 hour, and the absorbance of this solution was measured using a UV–vis spectrophotometer at 542 nm. The blood clotting index was evaluated from Eq. (S2):

$$\text{(2) Blood clotting index} = (A_{sample+blood}/A_{blood}) \times 100 \quad \text{(S2)}$$

Here, $A_{sample+blood}$ and A_{blood} refer to the absorbance of blood solutions that were in contact with the sample and diluted in 50 mL of DI water, respectively. The blood clotting test was repeated three times.

Acknowledgments

The financial support from Canakkale Onsekiz Mart University Scientific Research Commission (COMU-BAP: FBA-2022-4117) is greatly acknowledged.
